# Targeting the Renin–Angiotensin–Aldosterone System to Prevent Hypertension and Kidney Disease of Developmental Origins

**DOI:** 10.3390/ijms22052298

**Published:** 2021-02-25

**Authors:** Chien-Ning Hsu, You-Lin Tain

**Affiliations:** 1 Department of Pharmacy, Kaohsiung Chang Gung Memorial Hospital, Kaohsiung 833, Taiwan; cnhsu@cgmh.org.tw; 2 School of Pharmacy, Kaohsiung Medical University, Kaohsiung 807, Taiwan; 3 Department of Pediatrics, Kaohsiung Chang Gung Memorial Hospital and Chang Gung University College of Medicine, Kaohsiung 833, Taiwan; 4 Institute for Translational Research in Biomedicine, Kaohsiung Chang Gung Memorial Hospital and Chang Gung University College of Medicine, Kaohsiung 833, Taiwan

**Keywords:** chronic kidney disease, hypertension, renin-angiotensin-aldosterone system, nitric oxide, developmental origins of health and disease (DOHaD), oxidative stress, angiotensin-converting enzyme, nephron

## Abstract

The renin-angiotensin-aldosterone system (RAAS) is implicated in hypertension and kidney disease. The developing kidney can be programmed by various early-life insults by so-called renal programming, resulting in hypertension and kidney disease in adulthood. This theory is known as developmental origins of health and disease (DOHaD). Conversely, early RAAS-based interventions could reverse program processes to prevent a disease from occurring by so-called reprogramming. In the current review, we mainly summarize (1) the current knowledge on the RAAS implicated in renal programming; (2) current evidence supporting the connections between the aberrant RAAS and other mechanisms behind renal programming, such as oxidative stress, nitric oxide deficiency, epigenetic regulation, and gut microbiota dysbiosis; and (3) an overview of how RAAS-based reprogramming interventions may prevent hypertension and kidney disease of developmental origins. To accelerate the transition of RAAS-based interventions for prevention of hypertension and kidney disease, an extended comprehension of the RAAS implicated in renal programming is needed, as well as a greater focus on further clinical translation.

## 1. Introduction

Hypertension and chronic kidney disease (CKD) are highly prevalent diseases around the world. The WHO indicate that one in four men and one in five women have hypertension [1]. CKD affects up to ten percent of the world’s population [2]. Hypertension and CKD are closely interlinked [3], such that CKD is one of the most common causes of secondary hypertension and hypertension is an important factor related to CKD progression. The best-known example is renal artery stenosis, which is characterized by both hypertension and progressive loss of renal function [4]. It was recognized as the prototype of angiotensin-dependent hypertension, contributing to the discovery of the renin–angiotensin-aldosterone system (RAAS) [5].

A growing body of evidence suggests that both hypertension and kidney disease may have their origins in early life [6,7,8]. During kidney development, an exposure to a suboptimal intrauterine environment results in lifelong negative influences on renal structure and function and on renal compensatory mechanisms by so-called renal programming [9,10]. The developing kidney can be programmed by a diversity of early-life insults, leading to hypertension and kidney disease in adulthood. The concept that adverse conditions during organogenesis increase the vulnerability for developing adult diseases is called fetal origins hypothesis [11], more recently named “Developmental Origins of Health and Disease” (DOHaD) [12]. On the other hand, this concept leads to a theoretical shift of therapeutic approach from adult life to earlier stage, namely reprogramming, to potentially reverse disease processes before clinical disease becomes evident [13,14].

Blood pressure (BP) is tightly controlled by very complex networks, including the RAAS, endothelial function, sympathetic nervous system, natriuretic peptides, inflammation and the immune system [15,16,17]. The RAAS serves a counter-regulatory role in the pathogenesis and development of hypertension [17]. Several potential molecular mechanisms involved in developmental programming of hypertension and kidney disease have been addressed, including aberrant RAAS, oxidative stress, nitric oxide (NO) deficiency, gut microbiota dysbiosis, dysregulated nutrient-sensing signals, epigenetic regulation, and reduced nephron number [6,7,8,9,13,14,18,19,20]. Among them, the RAAS not only plays a vital role in the regulation of BP but also closely interacts with other mechanisms. The RAAS is a major hormone cascade composed of different angiotensin peptides with a variety of biological functions mediated by distinct receptors [21]. There are two major pathways in the RAAS: classical and non-classical pathways. The classical RAAS is mainly made up of angiotensin-converting enzyme (ACE), angiotensin (ANG) II, and angiotensin II type 1 receptor (AT1R). Under pathophysiological conditions, the classical RAAS can be activated to trigger vasoconstriction and inflammation, thus promoting hypertension and kidney damage [22]. Conversely, the non-classical RAAS composed of the ACE2-ANG-(1-7)-MAS receptor axis counterbalances the detrimental effects of ANG II signaling.

Of note is that both axes of the RAAS have been linked to fetal programming [23,24]. Although blockade of the classical RAAS provides the rationale for current antihypertensive and renoprotective therapies [25], there is limited data on whether early targeting on the RAAS can prevent hypertension and kidney disease of developmental origins. 

In the review, therefore, we present a contemporary update of the RAAS, explaining its role on hypertension and kidney disease of developmental origins and emphasizing its links to other mechanisms. We also highlight the potential reprogramming interventions that target the RAAS for prevention of developmental programming of hypertension and kidney disease. We retrieved related literature from all articles indexed in PubMed/MEDLINE. We used the following keywords and their combinations: “renin”, “angiotensin”, “chronic kidney disease”, “developmental programming”, “DOHaD”, “offspring”, “mother”, “nephrogenesis”, “nephron”, “prorenin receptor”, “aldosterone”, “mineralocorticoid receptor”, “pregnancy”, “progeny”, “reprogramming”, “angiotensinogen”, “angiotensin-converting enzyme”, and “hypertension”. Additional studies were then selected and evaluated based on appropriate references in eligible papers. The last search was conducted on 30 January 2021.

## 2. RAAS and the Programmed Kidney

### 2.1. Intrarenal RAAS 

The kidney is a principal target for the various components of the RAAS that include prorenin/renin, ANG II, ANG III (ANG-(2–8)), ANG-(1–7), ANG IV (ANG-(3–8)), ANG-(1–9), and aldosterone [26]. Renin starts a cascade of events in the RAAS. The kidney is the only known organ where prorenin to renin conversion occurs [27]. The substrate of the RAS, angiotensinogen (AGT) is released from the liver and is cleaved by renin to generate ANG I. ACE is universally existing in many cell types and tissues/organs. ACE is primarily known for its ability to cleave ANG I to ANG II, while it cleaves not only ANG I but also many other substrates including bradykinin [28]. ANG II stimulates the AT1R to enhance sodium reabsorption and elevate BP [29]. Conversely, ANG II type 2 receptor (AT2R) is the other type of ANG II receptors, which mediates vasodilatation. In the adrenal cortex, ANG II acts to cause the release of aldosterone. Aldosterone promotes sodium retention by stimulating sodium transporter in the distal tubules of the kidneys and, therefore, raises BP. Of note is that the renal RAAS is characterized by the highest tissue concentrations of ANG II [30]. In the kidney, ANG II can also be metabolized to ANG III (ANG-(2–8)) by aminopeptidase A (APA). In turn, ANG III is processed to ANG IV (ANG-(3–8)) by aminopeptidase N (APN) [30].

**Figure 1:**
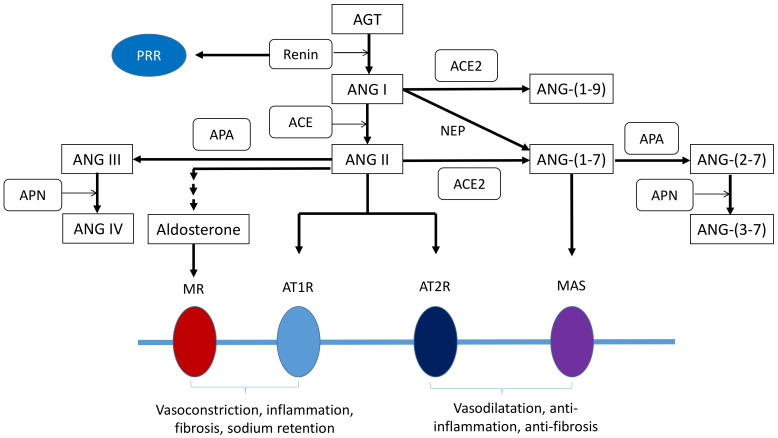
Schema outlining the renin-angiotensin-aldosterone system cascade including the renal effects of receptor stimulation. AGT, angiotensinogen; ACE, angiotensin-converting enzyme; ACE2, angiotensin-converting enzyme 2; ANG, angiotensin; ANG I, angiotensin I; ANG II, angiotensin II; ANG III, ANG-(2–8); ANG IV, ANG-(3–8); APA, aminopeptidase A; APN, aminopeptidase N; AT1R, angiotensin II type 1 receptor; AT2R, angiotensin II receptor; MAS, angiotensin-(1–7) receptor MAS; MR, mineralocorticoid receptor; NEP, neutral endopeptidase; PEP, prolyl endopeptidase; PRR, (Pro)renin- receptor.

On the other hand, ACE2, a homologue of ACE, which converts ANG II to ANG-(1–7) or converts ANG I to ANG-(1–9) [31]. ANG-(1–7) induces natriuretic and diuretic effects, in favor of vasodilatation via mediation of MAS receptor [24]. ANG I can also be converted to ANG-(1–7) by the endopeptidase neprilysin (NEP) [30]. In turn, ANG-(1–7) can be processed to (ANG-(2–7)) by APA, and further metabolized by APN to generate ANG-(3–7) [30]. Although most studies of the RAS have mainly focused on ANG II, other peptide fragments Ang-(1-7), ANG III (ANG-(2–8)), ANG IV (ANG-(3–8)), ANG-(2–7), and ANG-(3–7) were identified as potentially bioactive [30]. Since that different peptides in the RAAS could work in concert or in opposition, and that pharmacological alterations of the RAAS result in simultaneous changes of different ANG peptides and compensatory alterations in the abundance/activity of the participating RAAS enzymes, more extensive research work is necessary to understand the complexity of the network of RAAS peptides and how this network system affects renal programming. The processing of various ANG peptides in the RAAS in the kidney is illustrated in Figure 1.

### 2.2. The Programmed Kidney: Cause for Adult Hypertension and Kindey Disease?

The human kidneys are composed of nephrons ranged from 250,000 to 1.1 million per kidney [32]. Nephron is the functional unit of the kidney, but there is a wide variability with a 10-fold individual difference [32]. The formation of nephrons, namely nephrogenesis, commences at the 9th and continues until 36th week of gestation in humans [33]. The initiation of the kidney development takes place when a ureteric bud outgrowth from the nephric duct invades a group of mesenchymal cells contained within the caudal end of the nephric cord. The elaboration of the ureteric bud is known as branching morphogenesis [34], which leads to the formation of the nephrons and urinary collecting system. The key regulator of primary ureteric bud outgrowth and branching is glial-cell derived neurotrophic factor (GDNF) [35]. Nephron progenitors epithelialize to form the renal vesicle, which elongate to S-shaped body before fully developing into a nephron. There is an exponential increase in nephrons between 18 and 32 weeks. During the third trimester, nephron development is complete between the 32nd and 36th week of gestation [32]. Accordingly, normally nephrogenesis is complete at term. Premature infants thus likely have a reduced nephron endowment at birth. However, nephron number in preterm infants depends on not only gestational age, but also intrauterine environment and perinatal care. Impaired branching morphogenesis could cause low nephron endowment and a wide range of renal maldevelopment, namely congenital anomalies of the kidney and urinary tract (CAKUT). 

Important support for renal programming came from the Dutch famine birth cohort study, which revealed that malnutrition during gestation has long-lasting consequences for adult health, including hypertension and kidney disease [36,37]. Several epidemiologic studies have associated prematurity and low birth weight as risk factors for kidney disease and hypertension in later life [38,39,40]. Low birth weight can result from intrauterine growth restriction (IUGR) or preterm birth associated with low nephron number [32,33,41]. A reduced nephron number leads to compensatory glomerular hyperfiltration and glomerular hypertension. This starts a vicious cycle, with a further nephron loss that results in a rising BP, decline in renal function, and may end in CKD. 

Nevertheless, the number of nephrons cannot be determined in living humans. Although the use of ferritin-based nanoparticles as targeted magnetic resonance imaging (MRI) contrast agent to measure nephron number in human kidneys has made some progress [42], validation of a method for non-invasive in vivo assessment of nephron endowment deserves greater attention.

### 2.3. Impact of RAAS in Renal Programming

In the developing kidney, constituents of the RAAS are highly expressed and play a critical role in mediating proper renal morphology and physiological function [43,44]. In rats, all components of RAAS can be detected in the embryonic kidneys from 12 to 17 days of gestation, being higher in fetuses and newborn rats than in adult rats [44]. In humans, drugs interfering with the RAAS (e.g., ACE inhibitors [ACEIs] or angiotensin receptor blockers [ARBs]) have been avoided in pregnant women due to ACEI/ARB fetopathy and renal maldevelopment [45]. Prematurity was associated with an increase in plasma renin and ANG II levels, as well as ACE activity [46]. Animals lacking genes of the RAAS develop markedly abnormal kidneys [47,48]. On the other hand, animals transgenic for RAS genes display hypertension [49]. Blockade of the RAAS with ARB losartan during days 1 to 12 of postnatal life in the rat (during nephrogenesis stage) causes a reduced number of nephrons and hypertension in adulthood [50]. 

Some risk factors for developing hypertension and kidney disease were assessed in human studies. Nevertheless, these observational studies cannot per se directly establish a causal relationship between the early-life insults and adult disease. Additionally, these human studies do not illuminate molecular mechanisms by which hypertension and kidney disease are created and provide a reprogramming strategy. As a consequence of ethical considerations concerning what is feasible or not in human studies, animal models are of great importance. Given that human studies have many limitations, animal models were established to explore the types of insults driving renal programming, potential mechanisms of renal programming, the vulnerable periods during the kidney development, and potential reprogramming strategy.

## 3. Animal Models of Renal Programming: Impact of the RAAS

### 3.1. RAAS-Related Renal Programming in Animal Models

A growing number of animal models are now being established to study hypertension and kidney disease of developmental programming. As reviewed elsewhere [6,7,8,9,10,13,14,18,19,20], several environmental influences in early life that can program the kidney resulting in hypertension and kidney disease in later life, such as maternal malnutrition, maternal illness, maternal smoking, and exposure to medication or environmental toxins. Table 1 summarizes animal studies demonstrating the association between aberrant RAAS, early-life insults, and subsequent hypertension and kidney disease in adult offspring [51,52,53,54,55,56,57,58,59,60,61,62,63,64,65,66,67,68,69,70,71,72,73,74,75,76,77,78,79,80,81,82,83,84,85,86,87,88]. The present review is only restricted to environmental insults happening during the duration of nephrogenesis, with a focus on RAAS-related renal programming.

In this review, animal species range from rats [51,52,53,54,55,56,57,58,59,60,62,63,65,66,67,68,69,71,73,74,75,76,77,79,80,82,83,84,85,86,87], mice [61,72,78,81], rabbits [70], and sheep [64,88]. Rats and mice have been the dominant animal species used in research to study hypertension and kidney disease of developmental origins. Unlike human nephrogenesis, which is completed in utero, renal development in the rodent continues up to 2 weeks after birth [89]. Accordingly, environmental factors not only during pregnancy but also in early lactation period can impair renal development in rodents, resulting in renal programming and adult kidney disease. Table 1 demonstrates the outcomes evaluated in rats ranging from 4 to 90 weeks of age. As one human year equals to two rat weeks in adulthood [90], most outcomes evaluated are equal to human ages from infancy to middle adulthood. Nevertheless, essentially no information exists with regard to large animals to study the impact of RAAS on hypertension and kidney disease of developmental origin.

Table 1 indicates maternal malnutrition is the most common factor related to kidney disease and hypertension of developmental origins. A variety of nutritional insults can cause renal programming, including high sucrose consumption [51], high-fructose diet [52,53], protein restriction [58,59,60,61,62,63], calorie restriction [64], high-fat diet [65,66], high- salt diet [67], and low-salt intake [68]. Second, maternal illness is also interfering with renal programming. These medical conditions during pregnancy include hypertension [69,70], CKD [71], diabetes [72,73], chronodisruption [74], preeclampsia [75], infection [76], placenta insufficiency [77], and hypoxia [78]. Another factor disrupting renal programming is exposure to environmental chemicals or toxins, such as smoking [79,80], caffeine [81], and 2,3,7,8-tetrachlorodibenzo-p-dioxin (TCDD) [82]. Furthermore, renal programming can be triggered by medications like pyrrolidine dithiocarbamate [83] or glucocorticoid [84,85,86,87,88].

The most common adverse renal outcome of renal programming being studied is hypertension [51,52,53,54,55,56,57,58,59,60,61,62,63,64,65,67,68,69,70,71,72,73,74,75,76,77,78,79,80,81,82,83,84,85,86,87,88]. Albuminuria was demonstrated in offspring born of dams with protein restriction [58], diabetes [72], or hypoxia [78]. The glomerular filtration rate (GFR), an index of renal function, was reported as decreased [66,76], unaltered [54,55,59,60], or even increased [58] in different models of renal programming. Additionally, reduced nephron number [57,76,78,79], renal hypertrophy [71], glomerular hypertrophy [78], and tubulointerstitial injury [66,78] are major morphological deficits being reported. These observations indicate that the renal programming does not rely on one particular factor and it displays a wide range of phenotypes.

### 3.2. Renin, (Pro)renin, and Their Receptor in Renal Programming

Adverse renal outcomes are related to increased renin [53,74,75,78,81,83,85,86] and/or PRR [74,85] expression in most but not all animal models (see Table 1). Renin is mainly synthesized by the juxtaglomerular cells, located in the afferent arterioles of the kidney as preprorenin [91]. The signal peptide is cleaved off during transfer and generate prorenin. By cleavage of a 43-amino acid N-terminal fragment, prorenin is then converted to active renin. The kidney secretes both renin and prorenin into the circulation. Aside from cleaving AGT to generate ANG I, renin binds the PRR. This receptor also binds prorenin. The PRR protein is encoded for the *Atp6ap2* (ATPase 6 accessory protein 2). The PRR protein exists in three forms: (1) A full-length 35-39 kDa form consisting of 3 domains, (2) A 28 kDa soluble form, and (3) a truncated form [91].

As the RAAS cascade starts with renin, it raises the question of can we block the RAAS at its point of activation (i.e., renin) to prevent renal programming? The first selective renin inhibitor, aliskiren is noninferior to ACEIs and ARBs for BP reduction and was assessed as an efficient antihypertensive drug [92]. Aliskiren inhibits renin by binding to its catalytic site, thus inhibiting renin and prorenin activity, to block the RAS. However, renin and prorenin levels remain high, which could conceivably induce PRR signaling in an ANG II-independent manner.

Currently, PRR was identified for its multi-functional aspects, including (1) PRR enhances the RAAS by catalyzing ANG I production, (2) PRR induces mitogen-activated protein kinases (MAPK) signal pathway, (3) PRR is required as a subunit of V-ATPase, which transports protons across plasma membrane, and (4) PRR interacts with both the canonical Wnt/β-catenin and non-canonical Wnt/planar cell polarity (PCP) pathways, which are essential for embryonic development [91,93,94].

Many reports have shown that PRR signal pathway can induce ANG II-dependent hypertension [91]. Prorenin overexpression animals exhibited severe hypertension [95]. Unlike other RAS components, PRR knockout mice are lethal or, even tissue-specific, and have a short life expectancy [96], indicating a crucial function of PRR that is (pro)renin-independent. Yet there is currently little evidence about the role of ANG II-independent PRR signal transduction pathway on programmed hypertension.

**Figure 2:**
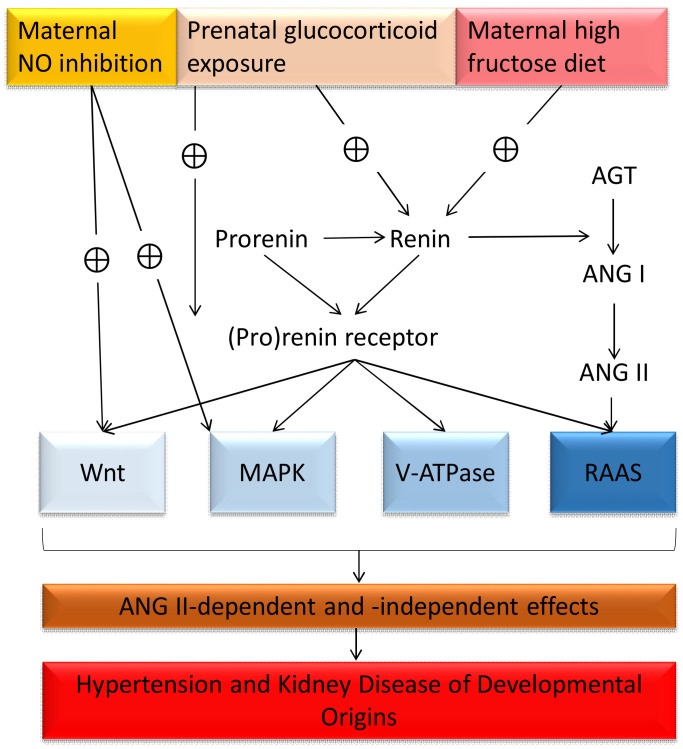
Flow diagram of identified pathways from previous models of renal programming, whereby (pro)renin–PRR pathway is linked to programmed hypertension and kidney disease via ANG II-dependent and –independent effects. AGT, angiotensinogen; ANG I, angiotensin I; ANG II, angiotensin II; MAPK, mitogen-activated protein kinases; NO, nitric oxide; RAAS, renin-angiotensin-aldosterone system, ⊕, enhance.

Our previous report showed that antenatal dexamethasone (DEX) administration increased renin (fold change = 2.41) and PRR (fold change = 2.37) mRNA expression during the stage of nephrogenesis [85]. The increase of renin expression was persistent until 4 months of age and was associated with elevated BP, indicating the impact of PRR on DEX-induced programmed hypertension. Next, we observed that maternal high-fructose increased renal renin expression from 1 day (fold change = 3.05) to 3 months (fold change = 3.38) of age [97]. These findings are consistent with previous studies showing the increases of plasma renin activity in offspring in a diversity of programming models [62,63,68]. Little reliable information currently exists with regard to PRR protein and its downstream signaling in animal models of renal programming. Whether decreased PRR expression could explain the absence of PRR-dependent effects during RAS inhibition remains to be further elucidated [94]. Furthermore, we observed that the downstream signal pathways of PRR, MAPK, and Wnt signal pathways were identified as the significant Kyoto Encyclopedia of Genes and Genomes (KEGG) pathways in the kidney of offspring using next generation RNA sequencing in an NO inhibition model [98]. All of these findings suggested that the PRR pathway might be a therapeutic target for programmed hypertension. The regulatory pathways related to PRR in different programming models are illustrated in the Figure 2.

### 3.3. Classical RAAS Axis in Renal Programming

Conflicting results exist regarding up- and downregulation of the classical RAAS components (Table 1), due in large part to the wide age range at which offspring were evaluated. In the majority of studies, adult offspring developed hypertension and kidney disease coinciding with increased expression of ACE [64,65,66,72,74,76,82,86] and AT1R [51,54,55,59,62,63,67,69,72,73,74,78,79,80,81,84], and ACE activity [73,77,88].

Very few studies have examined the RAAS in association with renal programming at different developmental stages. In a maternal low protein diet rat model [56], renal AT1R expression was suppressed at birth, whereas its expression was upregulated at 4 weeks of age. In another renal programming model induced by placental insufficiency in the Sprague Dawley rat, adult offspring developed hypertension in conjunction with increased renin and AGT mRNA, as well as increased ACE activity at 16 weeks of age [77]. Conversely, renin and AGT mRNA expression was decreased at birth [77]. Taken together, these findings in renal programming models suggested a transient biphasic response with downregulation of classical RAAS components in neonatal stage that becomes normalized with age. Various early-life insults may disturb this normalization in the adult, so much so that the classical RAS axis is inappropriately activated leading to the rising BP and development of kidney disease in adult offspring. Additionally, aberrant neonatal suppression of the intrarenal RAAS contributes to alterations of renal morphology [9], which is in agreement with studies reporting that blockade of the RAAS by ACEI or ARB [45].

It is noteworthy that aberrant activation of the RAAS can be transgenerational. In a maternal high-fructose diet model [99], elevation of BP was observed in the first- and second-generation offspring, with maximal increases in blood levels of renin, ANG II, and aldosterone in the third-generation offspring. Additionally, maternal high-fructose intake increased the renal mRNA expression of ACE and AT1R over multiple generations of offspring up until the third one. There will be a growing need to better understand whether transgenerational activation of the RAAS has potential impact on other models of renal programming.

### 3.4. Non-Classical RAAS Axis in Renal Programming

As the non-classical RAAS axis generally opposes the actions of the classical RAAS axis, a reduced tone of the ACE2-ANG-(1-7)-MAS receptor system is considered to contribute to those pathologies as well. Like classical RAAS axis, non-classical axis of the RAAS were also linked to fetal programming [24]. Table 1 shows adult offspring developed hypertension and kidney disease coinciding with downregulated non-classical RAAS pathway in several models of renal programming, including maternal low protein diet [61], maternal CKD [71], maternal diabetes [72], and glucocorticoid exposure model [87,88]. However, the reports were conflicting with increased ACE2 expression in the continuous light exposure model [74].

### 3.5. Aldosterone in Renal Programming

Aldosterone is the principal regulator of sodium homeostasis. The serum and glucocorticoid-regulated kinase isoform 1 (SGK1) is a key mediator of aldosterone action in the distal nephron to regulate almost all sodium transporters [100]. Compared to other components in the RAAS, less attention has been paid to evaluate the impact of aldosterone in animal models of renal programming. As shown in Table 1, only one report demonstrated that circulating aldosterone level was elevated in 8-week-old offspring born to dams exposed to a low-protein diet [57]. However, renal sodium transporters were studied in several models of renal programming, like antenatal glucocorticoid administration [84,101], low-protein diet [58,102], continuous light exposure model [74], and combined high-fructose and high-salt diet [103]. Various early-life insults have shown that renal programming is associated with increases mRNA levels and protein abundance of several sodium transporters like type 3 sodium hydrogen exchanger (NHE3), Na-K-2Cl cotransporter (NKCC2), Na+/K+ATPase α1 subunit (NaKATPase), and Na+/Cl− cotransporter (NCC). It is noteworthy that SGK1 can be activated by glucocorticoid and salt, except for aldosterone [104]. Therefore, if aberrant sodium transporters in above-mentioned animal models of renal programming are directly regulated by aldosterone or not awaits further clarification. Moreover, emerging evidence shows that fructose-induced hypertension is related to upregulation of the sodium transporter NHE3 and the chloride transporter putative anion transporter 1 (PAT1), to stimulate sodium and chloride absorption [105]. As much of previous work investigating the actions of RAAS has directly studied sodium transporters, there will be a need to better understand the interplay between the RAAS and chloride transporter in hypertension.

## 4. The Central Role of the RAAS on Mediating Common Mechanisms Underlying Renal Programming

**Figure 3:**
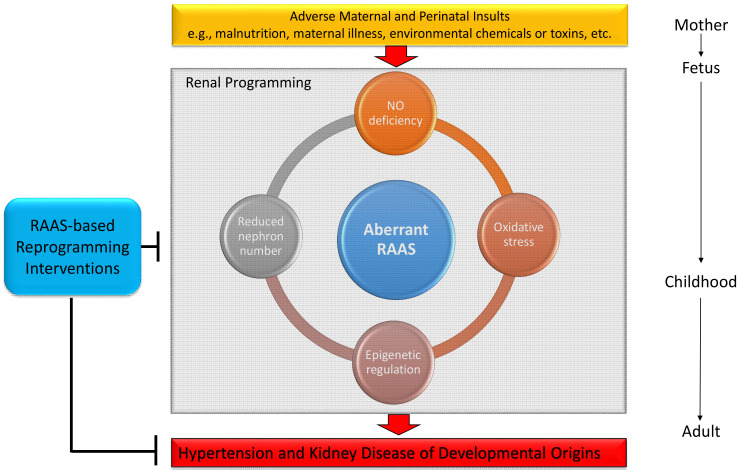
Schema outlining the central role of RAAS on mediating other mechanisms in the kidney leading to hypertension and kidney disease of developmental origins in response to a variety of maternal insults. Target on the RAAS-based interventions could be reprogramming strategies to prevent hypertension and kidney in adult offspring. NO, nitric oxide; RAAS, renin-angiotensin-aldosterone system.

In view of various early-life insults that elicit similar renal outcomes in adult offspring, there might be some common mechanisms of pathogenesis in renal programming. So far, several specific mechanisms were identified to explain renal programming. These mechanisms include aberrant RAAS, oxidative stress, nitric oxide (NO) deficiency, gut microbiota dysbiosis, dysregulated nutrient-sensing signals, epigenetic regulation, and reduced nephron number [6,7,8,9,10,13,18,19,20]. It is important to note that, among these proposed mechanisms, the RAAS is closely connected with others as a hub in determining the renal programming processes. The interplay between the RAAS and other proposed mechanisms underlying renal programming in response to adverse early-life insults is illustrated in Figure 3. Each mechanism will be discussed in turn.

### 4.1. Oxidative Stress

As reviewed elsewhere [106,107], the key role of oxidative stress implicated in hypertension and kidney disease of developmental origins is supported by many clinical and experimental studies. The imbalance of antioxidants defense system and the reactive oxygen species (ROS) production causes oxidative stress implicating fetal development [108]. Data from multiple animal models indicates oxidative stress involved in renal programming [106,107]. Among them, aberrant RAAS and oxidative stress are both associated with renal programming in models of prenatal DEX exposure [84], maternal high-fructose diet [53], high-fat diet [66], maternal CKD [71], preeclampsia [75], maternal TCDD and dexamethasone exposure [82], and prenatal DEX plus post-weaning high-fat diet [86]. It is well known that ANG II acting via AT1R is a potent activator of NADPH oxidase in the kidney, so much so that it enhances production of ROS implicating in the development of hypertension [109]. On the other hand, ROS-dependent enhancement of AGT plays a role in the progression of diabetic nephropathy [110].

In a model of renal programming, we observed inappropriate activation of the RAAS can be restored by antioxidant therapy [86]. Dimethyl fumarate (DMF) was reported to activate nuclear factor erythroid-derived 2-related factor 2 (Nrf2, a major player in the antioxidant defense) and protect against oxidative stress damage [111]. Our previous work showed DMF administration in pregnancy protects adult offspring against hypertension programmed by antenatal DEX plus postnatal high-fat diet, which was relevant to downregulated mRNA expression of renin, AGT, ACE, and AT1R [86]. Although clinical trials are utilizing Nrf2 inducers to treat CKD, Nrf2 activation was linked to unfavored effects like proteinuria and nephrogenic diabetes insipidus [112,113]. To what extent the Nrf2 activation can be beneficial on CKD, and how Nrf2 and oxidative stress are interconnected with the RAAS, are issues that await further clarification.

Another report showed that the protective effects of melatonin, a potent antioxidant, against programmed hypertension is attributed to increased renal ACE2 level [75]. Moreover, we previously examined the maternal light exposure-induced hypertension model and found maternal melatonin therapy protected offspring against hypertension coincided with increased renal ACE2 expression [74]. Also, melatonin therapy prevented the rise in offspring’s BPs coincided with increased ACE2 protein abundance in a maternal caloric restriction model [114]. These observations suggest that interplay between the RAAS and oxidative stress implicated in renal programming and consequently adverse renal outcomes.

### 4.2. Nitric Oxide Deficiency

The role of NO deficiency in mediating hypertension and kidney disease of developmental origins has received considerable attention [19,115]. One major cause of NO deficiency is due to increased asymmetric dimethylarginine (ADMA), an endogenous NOS inhibitor [116]. Targeting an ADMA/NO pathway to lower ADMA and restore NO was considered as a reprogramming approach to prevent renal programming and consequently hypertension and kidney disease [19,115].

ANG II can reduce NO bioavailability by promoting oxidative stress, while NO is able to counterbalance the vasoconstrictive effect of ANG II [117]. In a maternal L-N^G^-Nitro arginine methyl ester (L-NAME, an inhibitor of NO synthase) exposure model, NO depletion caused a rise in BP coinciding with increased mRNA expression of renin and ACE in offspring kidneys [75]. In another model of renal programming, blockade of the RAAS by aliskiren protected adult rat offspring against hypertension programmed by maternal caloric restriction in [118]. The protective effect of aliskiren is not only directed upon the RAAS, but also through regulation of the NO pathway, represented by decreases of plasma ADMA levels and increases of urinary NOx (NO_2_^-^+NO_3_^-^) levels [118]. Similar to renal programming models, early aliskiren therapy was reported to block the development of hypertension related to decreasing plasma ADMA levels in spontaneously hypertensive rats (SHRs), the most commonly used model of hypertension [119]. As the balance between ADMA/NO pathway and the RAAS plays a decisive role in the pathogenesis of renal programming, there will be a growing need to better understand the mechanisms of the actions of RAAS on renal programming, with a focus on its interplay with NO.

### 4.3. Reduced Nephron Number

A deficit in the number of nephrons causes high glomerular capillary pressure and glomerular hyperfiltration, consequently leading to further nephron loss in later life [8]. Accordingly, low nephron number was considered as a vital mechanism underlying renal programming. Several epidemiologic studies support that low birth weight and prematurity, two clinical surrogate markers of nephron number, are risk factors for adulthood hypertension and kidney disease [120,121,122]. In rats, adult offspring displayed reduced nephron number when DEX administration was for 2 days on embryonic day 13–14 or 17–18 [101]. These findings indicated the existence of developmental windows of vulnerability to environmental conditions during kidney development. As we mentioned earlier, blockade of the RAAS in lactation, the late stage of nephrogenesis in rodents, leads to reduced nephron number and hypertension in adulthood [50].

Several animal models of renal programming, as shown in Table 1, indicated that various adverse intrauterine conditions can lead to low nephron endowment and aberrant RAAS concurrently, as in the case of maternal protein restriction [56], maternal lipopolysaccharide (LPS) exposure [76], and prenatal hypoxia [78]. Prenatal hypoxia exposure resulted in a reduced nephron number by 25% and elevation of BP in male adult mice offspring, which is related to increases of renal mRNA expression of renin (~2-fold) and AT1R as well as renin concentrations (~50% increase) [78].

However, low nephron endowment, per se, is not essential for hypertension and kidney disease of developmental origins [8]. The roles of RAAS altering the nephron endowment behind the renal programming still remain to be identified, but are the subject of great interest.

### 4.4. Epigenetic Regulation

Epigenetic regulation is another important mechanism underlying fetal programming [123]. Epigenetic mechanisms consist of DNA methylation, histone modification, and non-coding RNAs (ncRNA). Global DNA methylation patterns in several organs were evaluated in different models of developmental programming, such as maternal low-protein diet [124], maternal smoking [125], and micronutrient deficiency [126]. However, less attention has been paid to the kidney. Aberrant DNA methylation was linked to hypertension of developmental origins [127]. In SHR, increased AT1R expression is relevant to progressive hypo-methylation in the AT1R promoter when hypertension occurs at 20 weeks of age [128]. However, the AT1R gene was reported to be hyper- or hypo-methylated in different models of programmed hypertension [23,129].

Additionally, epigenetic histone modification occurs when the N-terminal tail is subjected to a diversity of post-translational modifications [130]. One of the most frequent epigenetic modifications is histone acetylation, which is catalyzed by histone acetyltransferases (HATs). Conversely, histone deacetylases (HDACs) determine histone deacetylation. The crosstalk between HDAC and the RAAS was proposed to drive uretic bud branching during kidney development [18]. HDACs were reported for the regulated expression of several genes belonging to the RAAS, including AGT, renin, ACE, and AT1R [131]. Our previous study showed that trichostatin A, a HDAC inhibitor, prevented neonatal DEX-induced programmed hypertension accompanied with decreases of AGT, ACE, and ACE2 [87].

The ncRNAs are implicated in several epigenetic processes [132], and microRNAs (miRNAs) are the most commonly studied small ncRNA. In regards to the RAAS-regulated genes, analysis of miRNA binding sites by TargetScan [133] suggested that 368 different miRNA families target RAAS elements, the majority of which share transcripts. In a maternal protein restriction model, renal epithelial-to-mesenchymal transition was associated with a reduced level of miR-200a, miR-141, and miR-429 [134]. Another report demonstrated that mmu-miR-27a and mmu-miR-27b upregulated ACE, while mmu-mir-330 downregulated AT2R in offspring born to dams with protein restriction [135]. However, a single miRNA can regulate numerous mRNAs makes it more challenging to decipher the exact mechanisms involved in renal programming. Additional human and experimental studies are required to clarify the exact nature of the mechanisms behind and to develop potential therapeutic applications.

### 4.5. Others

There are other reported mechanisms behind renal programming by which the RAAS might act: (1) dysregulated nutrient-sensing signals, (2) gut microbiota dysbiosis, and (3) sex differences. First, early-life nutritional insults can impair nutrient-sensing signals that affect fetal development and consequently program hypertension in later life [136]. Peroxisome proliferator-activated receptor (PPAR), one of the nutrient-sensing signals, can be mediated by other nutrient-sensing signals to regulate the expression of PPAR target genes [137]. Of note is that several PPAR target genes belong to the RAAS components or sodium transporters, like renin and SGK1 [138]. As reviewed elsewhere [138], emerging evidence has indicated that early intervention by PPAR modulators can prevent hypertension of developmental origins. Thus, it is speculated that the RAAS may interact with nutrient-sensing signals to program hypertension and kidney disease.

Second, adverse intrauterine conditions can disturb the gut microbial balance, resulting in subsequent adverse offspring outcomes, including hypertension [139]. Prior research showed that ACE2 exerts a non-catalytic role in gut biology and modulates gut microbiota composition [140]. As dysbiosis of the gut microbiome has been linked to hypertension by modulating the gut RAAS [141], these findings suggested there might be a relationship between gut microbiota and the RAAS underlying the pathogenesis of renal programming, although this remains speculative.

Last, emerging evidence supports sex-dependent differences exist in hypertension and kidney disease of developmental origins [142,143]. It is noteworthy that the RAAS was reported as a sex-specific response to environmental insults [144]. Also, an alteration in the response of the renal transcriptome to diverse insults is sex-dependent [52,145,146]. However, much of the animal models of renal programming, as shown in Table 1, mainly investigating males only instead of both sexes. Thus, there will be a growing need to elucidate the impact of RAAS on sex-dependent mechanisms behind renal programming, and to be able to develop novel sex-specific strategies targeting the RAAS to prevent programmed hypertension and kidney disease of developmental origins in both sexes.

Although the multiple mechanistic links outlined above, the RAAS works as a central connection for hypertension and kidney disease of developmental origins. Better understanding interaction between the RAAS and other common mechanisms as well as targeting on the RAAS to develop reprogramming intervention are key toward early prevention or treating prehypertension and subclinical kidney disease.

## 5. Targeting on the RAAS as Reprogramming Strategies

Reprogramming strategies targeting the RAAS to prevent the developmental programming of hypertension and kidney disease that were employed in various animal models are listed in Table 2 [52,63,118,119,147,148,149,150,151,152,153,154]. Currently, several therapeutic interventions have been reported, such as renin inhibitor [52,118,119], ACEI [63,147,148,149,150], ARB [118,151,152], AT1R antisense [153], and ACE2 activator [154]. The major protective effects of various RAAS-based interventions on adverse renal outcomes are against hypertension [52,63,118,119,147,148,149,150,151,152,153,154], followed by albuminuria [149], renal dysfunction [150], and renal fibrosis [154]. The reprogramming effects of RAAS-based therapies were examined in rats ranging from 9 weeks to 6 months of age, which are almost equivalent to human ages from childhood to young adulthood. However, most studies focused on males only and did not test different doses. Whether these observed effects appear in a dose- or sex-dependent manner awaits further studies for clarification.

Early blockade of the classical RAAS axis was proposed to reprogram the inappropriately activated RAAS to prevent hypertension and kidney disease of developmental origins. Treating the young offspring with renin inhibitor aliskiren [52,118], ACEI captopril [63], or ARB losartan [118,151] between 2–4 weeks of age are most common therapeutic periods to offset the effects of developmental programming on BP.

To date, aliskiren is the only one renin inhibitor approved for treating hypertension. However, aliskiren cannot prevent the interaction between the PRR and its ligand. Even though beneficial effects for the PRR inhibitory peptide, handle region peptide, and PRO20 [155,156] were reported in animal models, the efficacy in specificity of these peptides is questionable [94]. Thus, it is hoped that designing a specific non-peptide inhibitor of PRR could result in favorable (pro)renin–PRR inhibition in the near future.

All of the prior work investigating reprogramming interventions only studied rats. As nephrogenesis is completed in the second postnatal week in the rat, almost all RAAS-blockade interventions to prevent the hypertension and kidney disease start as early as two weeks after birth. Although AT1R antisense delivery was performed at postnatal day 5 in SHRs [153], its effect on nephron number was not examined yet.

Apart from the classical axis in the RAAS, emerging evidence provides protective roles of non-classical axis in established hypertension and kidney disease, paving the way for new therapeutic approaches [21]. Nevertheless, little attention has been paid to apply this approach on programmed hypertension and kidney disease. According to Table 2, only one study reported administration with diminazene aceturate (DIZE), a putative ACE2 activator, or with ANG -(1–7) during pregnancy could attenuate hypertension and renal fibrosis in adult SHR offspring [154]. Owing to activation of ACE2-ANG-(1-7)-MA axis having therapeutic potential in established hypertension and kidney disease, there is an ongoing need for additional study to elucidate its reprogramming effects in renal programming. What is missing from the literature is a deeper understanding of which the most important component of the RAAS is for the targeted approach and what time is the optimal therapeutic window to be used to prevent hypertension and kidney disease of developmental origins.

## 6. Conclusions

Current evidence has provided vigorous but incomplete data in regard to the potential therapeutic role of RAAS-based interventions in hypertension and kidney disease of developmental origins. This review affords a brief overview on the various RAAS-based therapies that shows benefits on renal programming, including renin inhibitor, ACEI, ARB, AT1R antisense, and ACE2 activator.

So far, one major unsolved problem is that almost no studies have taken a holistic approach to simultaneous quantify the expression/activity of the entire repertoire of the RAAS components in an experiment. Due to the complex nature of RAAS signaling, the reprogramming effect in response to early-life RAAS-based interventions, either individually or in combination, are incomplete and difficult to predict. Therefore, future work in developing ideal methodology is needed to get a more holistic view of the RAAS and ensure RAAS-based therapy would only apply in the right direction. Moreover, attention will need to be paid to decide the optimal dosage in a sex-dependent manner to maximize the benefit without increasing toxicity prior to clinical translation.

Despite significant progress being made in the availability of a broad range of RAAS-based drugs, less attention has been paid to investigate their reprogramming effects on hypertension and kidney disease. Another challenge is that specific developmental windows for different RAAS-based therapies to reprogram the processes driving hypertension and kidney disease still await further clarification.

For now, our review has taken a step forward by linking RAAS to hypertension and kidney disease of developmental origins, which may yield insights into new RAAS-based interventions for preventing renal programming-related disorders in a clinical setting.

## Figures and Tables

**Table 1:** Renal programming related to aberrant renin-angiotensin-aldosterone system (RAAS) in animal models. Studies tabulated according to animal models, species, and age at evaluation. CKD, chronic kidney disease; L-NAME, L-N^G^-Nitro arginine methyl ester; LPS, lipopolysaccharide; TCDD, 2,3,7,8-tetrachlorodibenzo-p-dioxin; PDTC, pyrrolidine dithiocarbamate; DEX, dexamethasone; Cr, creatinine; SD, Sprague Dawley; SHR, spontaneously hypertensive rat; M, male; F, female; BP, blood pressure; GFR, glomerular filtration rate; ↑, increased; ↓, decreased; ↔, unaltered; PRR, (pro)renin receptor; PRA, plasma renin activity; ACE, angiotensin-converting enzyme; ACE2, angiotensin-converting enzyme 2; AGT, angiotensinogen; AT1R, angiotensin type 1 receptor; AT2R, angiotensin type 2 receptor.

Animal Models	Intervention Period	Species/Gender	Age at Evaluation	Renal Phenotype	Alterations of the RAAS	Ref.
20% *w/v* sucrose in drinking water	Pregnancy	SD rat/M	90 weeks	↑BP	↑AT1R mRNA and protein	[51]
High-fructose diet, 60%	Pregnancy and Lactation	SD rat/M	12 weeks	↑BP	↓AT2R mRNA	[52]
High-fructose diet, 60%	Pregnancy and Lactation	SD rat/M	12 weeks	↑BP, altered renal transcriptome	↑Renin mRNA	[53]
Protein restriction, 9%	Pregnancy	SD rat/M	4 weeks	↑BP, ↔GFR	↑AT1R protein and ↓AT2R protein, ↔ANG level	[54,55]
Protein restriction, 6%	Pregnancy	SD rat/M and F	4 weeks	↑BP	↓AT1R and AT2R protein at birth; ↑AT1R and AT2R protein at 4 wk	[56]
Protein restriction, 6%	Pregnancy	SD rat/M and F	8 weeks	↑BP, ↓nephron number	↓PRA, ↓AT1R mRNA and protein, ↑Aldosterone	[57]
Protein restriction, 8%	Lactation	Wistar rat/M	150 days	↑BP, ↑GFR, ↑Proteinuria	↑AT1R protein and↓AT2R protein	[58]
Protein restriction, 8.5%	Pregnancy	SD rat/M	22 weeks	↑BP, ↔GFR	↓renin mRNA and protein; and ↓renal ANG II level at 1–5 days of age	[59,60]
Protein restriction, 9%	1 week before conception and throughout pregnancy	FVB/NJ mouse/F	24 weeks	↑BP	↓ACE2 protein	[61]
Protein restriction	Second half of pregnancy	SD and Wistar rat/M and F	11 months	↑BP	↑PRA, ↑AT1R mRNA and protein	[62,63]
50% caloric restriction	Day 28 to day 78 of gestation	Sheep/M and F	9 months	↑BP	↑ACE protein	[64]
High-fat diet, 58%	Pregnancy and Lactation	SD rat/M	16 weeks	↑BP	↑AGT and ACE mRNA, and↑AT1R protein	[65]
High-fat diet, 58%	5 weeks before the delivery and throughout pregnancy and lactation	SD rat/M and F	6 months	↔BP, ↓GFR, ↑glomerular injury, ↑tubulointerstitial injury, altered renal transcriptome	↑ACE and AT1R mRNA in F	[66]
High-salt diet, 8%	Pregnancy	Wistar rat/F	12 weeks	↑BP	↑ANG II	[67]
0.03% low-salt diet	Last 7 days of pregnancy	SD rat/M and F	12 weeks	↑BP	↑PRA	[68]
Maternal renovascular hypertension	Pregnancy	SD rat/M	16 weeks	↑BP	↑AT1R protein	[69]
Maternal renovascular hypertension	Pregnancy	Rabbit/F	30 weeks	↑BP	↓PRA at 10 week	[70]
Maternal adenine-induced CKD	Pregnancy and lactation	SD rat/M	12 weeks	↑BP, renal hypertrophy	↓AT2R and MAS receptor mRNA	[71]
Maternal streptozotocin-induced diabetes	Pregnancy	C57BL/6 mouse/M	20 weeks	↑BP, microalbuminuria	↑AT1R and ACE mRNA, ↓ACE2 mRNA	[72]
Maternal streptozotocin-induced diabetes	Pregnancy	Wistar rat/M	2 months	↑BP	↑ACE activity	[73]
Continuous light exposure	Pregnancy and lactation	SD rat/M	12 weeks	↑BP	↑Renin, PRR, AGT, ACE, ACE2, and AT1R mRNA	[74]
Maternal L-NAME exposure	Pregnancy	SD rat/M	12 weeks	↑BP	↑Renin and ACE mRNA	[75]
Maternal LPS exposure	Pregnancy	SD rat/M and F	24 weeks	↑BP, ↓nephron number and GFR	↑ACE mRNA	[76]
Placenta insufficiency	Pregnancy	SD rat/M	16 weeks	↑BP	↓Renin and AGT mRNA at birth, ↑Renin and AGT mRNA, ↑ACE activity at 16 week	[77]
Prenatal hypoxia	From embryonic day 14.5 until birth	CD1 mouse/M and F	12 months	Microalbuminuria, glomerular hypertrophy and renal fibrosis, ↓nephron number	↑Renin and AT1R mRNA	[78]
Maternal nicotine exposure	Pregnancy	SHR/M	9 weeks	↑BP, ↓Glomerular mass	↑AT1R mRNA	[79]
Maternal nicotine exposure	Pregnancy	SD rat/M	5 months	↑BP	↑AT1R protein, ↓AT2R protein	[80]
Maternal caffeine exposure	Pregnancy	C57BL/6 mouse/M	3 months	↑BP	↑Renin and AT1R mRNA	[81]
Maternal TCDD and dexamethasone exposure	Pregnancy and Lactation	SD rat/M	16 weeks	↑BP	↑ACE mRNA	[82]
Neonatal PDTC administration	Lactation	Munich-Wistar rat/M	10 months	↑BP	↑Renin and AGT mRNA at 3 month; ↓Renin and AGT at 10 month	[83]
Prenstal DEX exposure	Pregnancy	SD rat/M	16 weeks	↑BP	↑AGT and AT1R mRNA	[84]
Prenstal DEX exposure	Pregnancy	SD rat/M	16 weeks	↑BP	↑Renin and PRR mRNA	[85]
Prenatal DEX plus post-weaning high-fat diet	Pregnancy	SD rat/M	16 weeks	↑BP	↑Renin and ACE mRNA	[86]
Neonatal DEX administration	Day 1 to day 3 after birth	SD rat/M	16 weeks	↑BP	↓AGT, ACE, and ACE2 mRNA	[87]
Prenatal betamethasone exposure	2 doses, 24 h apart at gestational day 80	Sheep/M	1.8 years	↑BP	↑ACE activity, ↓ACE2 activity	[88]

**Table 2:** Interventions targeting on the RAAS to prevent hypertension and kidney disease of developmental origins.Studies tabulated according to types of intervention, animal model, species, and age at evaluation. SD, Sprague Dawley; SHR, spontaneously hypertensive rat; M, male; F, female; ACE2, angiotensin-converting enzyme 2; AGT, angiotensinogen; Ang II, angiotensin II; AT1R, angiotensin type 1 receptor; MAS receptor, ANG-(1–7) receptor MAS.

Intervention	Animal Model	Species/Gender	Age at Evaluation	Effects	Protective Mechanism	Ref.
Renin inhibitor						
Aliskiren (10 or 30 mg/kg/day) between 4–10 weeks of age	Genetic hypertension model	SHR/M	10 weeks	Prevented or attenuated hypertension by 30 or 10 mg, respectively	Restoration of NO bioavailability	[118]
Aliskiren (10 mg/kg/day) between 2–4 weeks of age	Maternal 50% caloric restriction	SD rat/M	12 weeks	Prevented hypertension	Decreased renal AGT mRNA; Increased renal ACE2 and MAS receptor protein levels	[119]
Aliskiren (10 mg/kg/day) between 2–4 weeks of age	Maternal high-fructose diet	SD rat/M and F	12 weeks	Prevented hypertension in both sexes	Increased renal ACE2 and MAS receptor protein levels in F	[52]
ACEI						
Captopril (100 mg/kg/day) between 2–4 weeks of age	Maternal protein restriction	Wistar rat/M	12 weeks	Prevented hypertension	Not evaluated	[63]
Captopril (100 mg/kg b.w./day) between 4–10 weeks of age	Genetic hypertension model	SHR/M	30 weeks	Attenuated hypertension	Not evaluated	[147]
Enalapril (100 mg/L) in drinking water between 3–6 weeks of age	Maternal protein restriction	SD rat/M	16 weeks	Prevented hypertension	Not evaluated	[148]
Enalapril (100 mg/L) in drinking water between 3–6 weeks of age	Maternal protein restriction	SD rat/M	6 months	Prevented hypertension and albuminuria	Reduced urinary AGT and ANG II levels	[149]
Perindopril (3 mg/kg/day) between 4–16 weeks of age	Genetic hypertension model	SHR/M	28 weeks	Attenuated hypertension and renal dysfunction	Not evaluated	[150]
ARB						
Losartan (100 mg/L) in drinking water between 2–4 weeks of age	Maternal protein restriction	Wistar rat/M	12 weeks	Prevented hypertension	Not evaluated	[151]
Losartan (20 mg/kg/day) between 2–4 weeks of age	Maternal 50% caloric restriction	SD rat/M	12 weeks	Prevented hypertension	Decreased renal AGT mRNA	[118]
Losartan (20 mg/kg/day) between 4–9 weeks of age	Genetic hypertension model	SHR/M	9 weeks	Prevented hypertension	Increased renal ACE2 expression	[152]
AT1R antisense						
AT1R antisense delivery at 5 days of age	Genetic hypertension model	SHR/M	3 months	Prevented hypertension	Decreased AT1R mRNA	[153]
ACE2 activator						
Diminazene aceturate in pregnancy	Maternal hypertension	SHR/M	16 weeks	Attenuated hypertension and renal fibrosis	Not evaluated in the kidney	[154]
ANG-(1-7) in pregnancy	Maternal hypertension	SHR/M	16 weeks	Attenuated hypertension and renal fibrosis	Not evaluated in the kidney	[154]
